# The uses and gratifications of social media and their impact on social relationships and psychological well-being

**DOI:** 10.3389/fpsyt.2024.1260565

**Published:** 2024-03-04

**Authors:** Veera Bhatiasevi

**Affiliations:** Mahidol University International College, Mahidol University, Nakhon Pathom, Thailand

**Keywords:** social media use, adults, older adults, uses and gratifications theory, social relationships, psychological well-being, Thailand

## Abstract

This study aims to find the antecedents that lead to the adoption of social media among adults and older adults in Thailand and the impact it has on their social relationships and psychological well-being. It puts forward the uses and gratifications theory focusing on purposive value, self-discovery, entertainment value, social enhancement, and maintaining interpersonal connectivity. A survey comprising of 1,176 participants was undertaken in Bangkok, Thailand. The results of the structural equation modeling show that purposive value, entertainment value, social enhancement, and maintaining interpersonal connectivity had a positive relationship with social media usage, while self-discovery showed a negative relationship. Social media use seemed to positively affect both the social relationships and psychological well-being of their users. The discussions and conclusions included here describe how this occurs, as well as the academic and practical implications that follow from them.

## 1 Introduction

Information Technology (IT) has become one of the most essential elements affecting the way people live and work. The invention of the World Wide Web by Tim-Bernes Lee in 1991 allowed for hypertext technology to interconnect via the Internet. This networked communication was rather generic, allowing users to merely create groups but not automatically connect to other users (1). The introduction of Web 2.0 saw a shift of communication between users through social media platforms, which has led to an increase in virtual communications (2). The Pew Research Center (3) also noted that social media can serve and enable people to gain various benefits from searching for information, sharing experiences, and strengthening bonds and relationships between family and friends.

**Figure 1:**
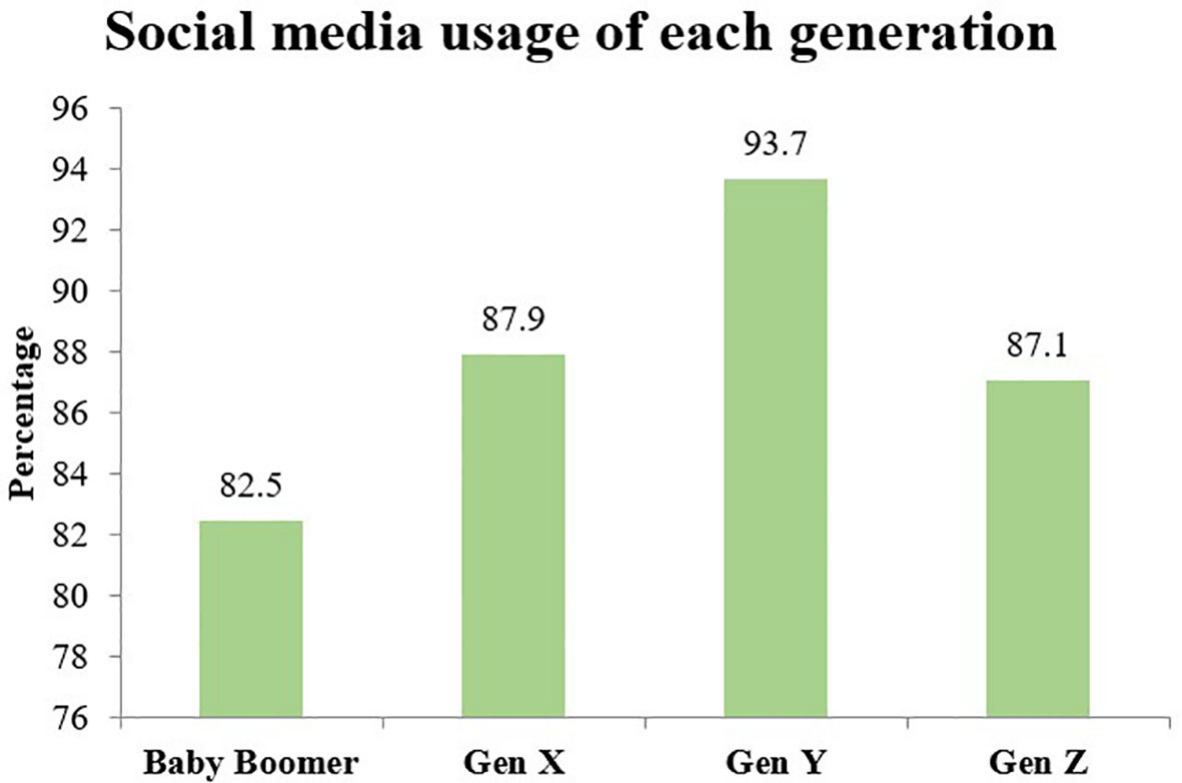
Social media usage by generation. (Source: (5)).

**Figure 2:**
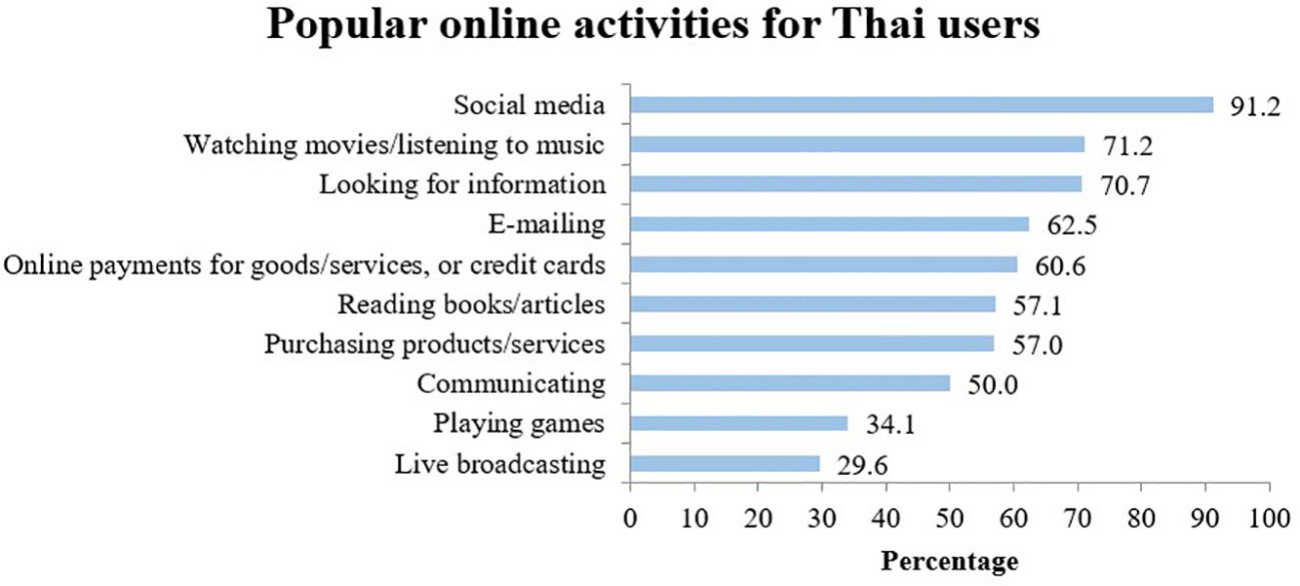
Popular online activities for Thai users. (Source: (5)).

As of 2020, there were 52 million Internet users in Thailand compared to 26.14 million a decade earlier, signaling an increase in usage by almost 100 percent (4). On average, Thais spend 10.22 hours per day on the Internet, a steep rise from 4.36 hours a day in 2013, with differences among generations in terms of usage behavior relatively slight. For example, baby boomers go online 10 hours per day, Generation X, 9.49 hours per day, Generation Y, 10.36 hours per day, and Generation Z, 10.36 hours per day (5). While there is some difference between the different generations with regard to how they use social media, with Generation Y being most active users, as shown in **Figure 1**, It is nonetheless evident that social media use is popular across all four generations. **Figure 2** shows that the use of social media is the most popular online activity by Thai users.

**Figure 3:**
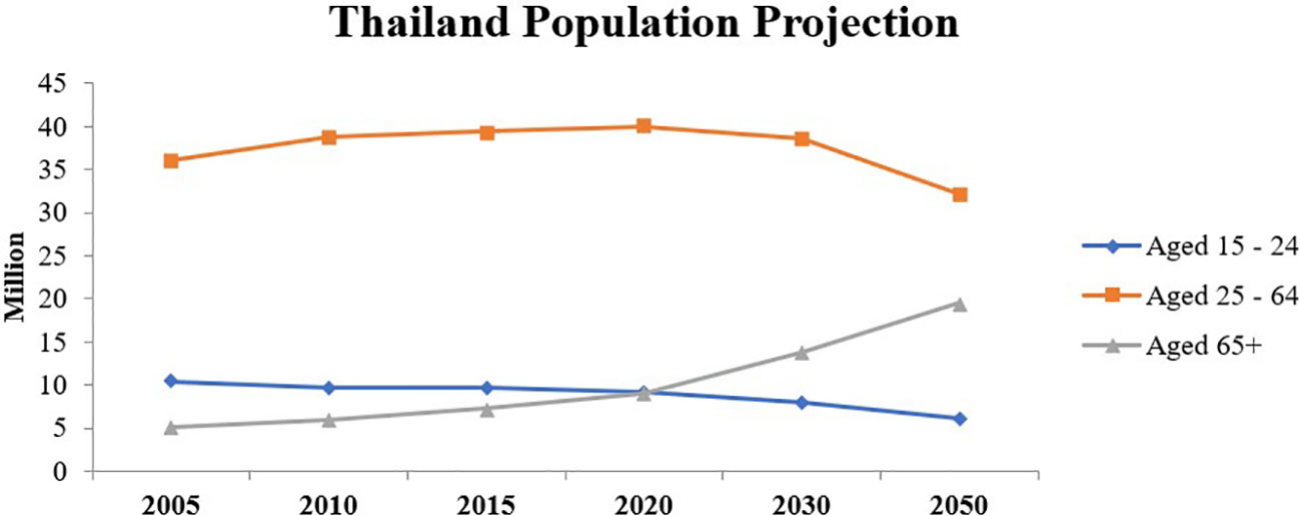
Thailand population projection. (Source: 6).

The high percentages of Internet and social media use among both younger adults and older adults and the shift of the population landscape towards older adults (as shown in **Figure 3**) indicate that it is imperative to know the factors leading to their use of social media and how it effects their social relationships and psychological well-being. Therefore, the objectives of this research are:

1. investigating the antecedents that lead to social media adoption among both younger and older adults*^1^
2. identifying the extent to which each antecedent has on social media adoption among both younger and older adults
3. investigating the effects that social media use has on the social relationships and psychological well-being of both younger adults and older adults

**Table 1** presents a list of studies that have been conducted in the field of social media use and the elderly, indicating that this topic has received a lot of interest among researchers. However, an extensive literature review shows no studies measuring the impact on social relationships and psychological well-being, especially in the context of an emerging economy like Thailand. Examining social media use and its effects in Thailand can provides a comprehensive framework for understanding the effects social media adoption as well as providing empirical evidence and insight for social media application developers as well as related government agencies.

**Table 1:** Summary of previous studies on social media use among the elderly.

Authors	Topic	Methods	Country
He et al. (2020) (7)	Social participation of the elderly in China: The roles of conventional media, digital access and social media engagement	Quantitative survey (n=1,399)	China
Wu and Chiou (2020) (8)	Social media usage, social support, intergenerational relationships, and depressive symptoms among older adults	Quantitative survey (n=153)	Taiwan
Pan et al. (2018) (9)	Social participation among older adults in Belgium’s Flanders region: Exploring the roles of both new and old media usage	Quantitative survey (n=36,282)	Belgium
Teng and Joo (2017) (10)	Analyzing the usage of social media: A study on elderly in Malaysia	Quantitative survey (n=200)	Malaysia
Parida et al. (2016) (11)	Factors for elderly use of social media for health‐related activities	Quantitative survey (n=610)	Sweden
Hutto et al. (2015) (12)	Social media gerontology: Understanding social media usage among older adults	Quantitative survey (n=268)	United States

## 2 Literature review and research model

### 2.1 Social media use

Since its inception, social media and social networks have been defined by different scholars to reflect various aspects associated with it. According to Cotten et al. (13), social media can be defined *“as a range of platforms, including social networking sites, virtual communities, blogs, social gaming, video sharing, and so forth, that allow users to share content and connect and interact with others online”* (p.1). From this definition, it can be inferred that the term social media covers a range of devices and platforms. The popularity of social media has made it ubiquitous and a part of the daily life of many users (14, 15).

Southeast Asia ranks second in the world, only behind East Asia in terms of social media users (16), and Thailand ranks seventh overall in Asia and fourth in Southeast Asia in terms of active social networks penetration, behind Singapore, Malaysia, and the Philippines. (17). The statistics provided indicate that social media use is indeed very popular in Thailand, where Facebook, YouTube, and Line are the three most popular social media platforms (18).

### 2.2 Uses and gratifications theory

The uses and gratifications theory (UGT), introduced by Katz et al. (19), is a sociological theory that describes the reasoning behind persons’ selection and use of media to satisfy their specific needs. They explained that people’s needs could be categorized as social and psychological, including cognitive, affective, integrative, social integrative, and escape needs. The theory focuses more on the how and why, which indicates that people are goal oriented in the media they select (19). Therefore, when they use a particular medium to satisfy their specific needs they also derive a certain level of satisfaction from using it (20). West and Turner (21) also confirmed this, noting that UGT assumes that users have already realized their objectives and needs; therefore, users will evaluate the value of the content presented by the media in accordance with their needs and satisfactions. Valentine (22) suggested that UGT explains the three steps for the user and the use of a certain media, namely: motives for media use, factors influencing these motives, and the outcomes of using the media.

In the past, UGT has been applied to traditional media such as newspapers, radio, and television. Examples include Towers’ (23) study on UGT and newspaper readership, UGT and radio listenership (24), and Kang and Atkin’s (25) study on UGT and multimedia cable adoption. However, since the advent of the Internet, UGT has also been applied to various types of digital platforms—for example, to help understand the moderating role of E-WoM and traditional media advertisement in fast-food joint selection (26), to learn what attracts consumers to local retail platforms (27), to understand the impacts of gamification designs on consumer purchases (28), to examine the impact of excessive Instagram use on students’ academic studies (29), to assess augmented reality (AR) applications and whether these can enhance students’ experiences (30), and to learn more about purchasing and continuation intentions with regard to over-the-top video streaming platform subscriptions (31).

Other studies have used UGT to focus on how social media operates in value cocreation behavior (32), how platforms such as e-Tutor employ Facebook for informal learning (33), how social media live streams affect online buyers (34), how users’ continuous content contribution behavior functions on microblogs (35), how privacy is treated on online social networks (15), and how the adoption of social networking sites has become so pervasive (36). Therefore, it is evident that UGT can be applied to the adoption of various forms of technology.

Various adaptations of UGT have also been applied in previous studies. Gan and Li (37) used UGT to understand how gratification fostered the intention to continue to use WeChat, Kamboj (38) used UGT to understand how gratification can be obtained in the context of social networking services through relationship building, information seeking, entertainment, brand likeability, and incentivization, while Liu et al. (35) used UGT to understand users’ expectations towards self-expression, anticipated extrinsic rewards, and anticipated reciprocity. In this study, Ifinedo’s (36) five categories of UGT will be used. They are: purposive value, self-discovery, entertainment value, social enhancement, and interpersonal connectivity. These five categories were pioneered by Dholakia et al. (39) in their study of individual participation in online communities. Subsequently, this UGT framework was also adopted by Raza et al. (40). James et al. (41), and Cheung and Lee (14).

### 2.3 Uses and gratification theory and usage behavior

According to Dholakia et al. (39) purposive value is defined as *“the value derived from accomplishing some pre-determined instrumental purpose (including giving or receiving information) through virtual community participation”* (p.244). Previous studies have shown that users use social media for a purpose. Hossain (42) states that users use Facebook to share information, videos, and photos as well as to download and upload information to groups with which they share common interests, while Gulzar et al. (43) conclude that *“social media is used for a variety of purposes, including messaging, emailing, knowledge sharing, chatting, advertising, buying and selling, booking of airlines and hotels, and studying”* (p.2283). Thus, users tend to use social media to find information, generate ideas, provide others with their information, or solve problems. Therefore, the following hypothesis is proposed:

H1. Purposive value has a positive relation with the use of social media.

Dholakia et al. (39) defined self-discovery as “*understanding and deepening salient aspects of yourself through social interactions, such interactions may help one to form, clearly define and elaborate on one’s own preferences, tastes and values”* (p.244). Ifinedo’s (36) examination of social influence processes to understand students’ pervasive adoption of social networking sites concluded that self-discovery has a positive influence on the use of social networking sites. Jin et al. (44) found similar factors affecting users’ intentions to continue participating in virtual communities. Thus, users will use social media if they feel that they are able to learn more about themselves, gain insights, and have a better understanding of themselves. In light of this, the following hypothesis is proposed:

H2. Self-discovery has a positive relation with the use of social media.

According to Dholakia et al. (39), entertainment value, is *“derived from fun and relaxation through games or otherwise interacting with others”* (p.244). Previous studies have found a positive relationship between the use of social media for entertainment (40) on the influence of social networking sites on life satisfaction (45), on the effect of social influence, trust, and entertainment value on social media use (46), on users’ engagement with Facebook, and on life satisfaction derived from using Facebook (47). Users will use social media if they feel that they are having fun, derive a sense of pleasure from using it, and do not feel bored from using it. Therefore, the following hypothesis is proposed:

H3. Entertainment value has a positive relation with the use of social media.

Dholakia et al. (39) defined social enhancement as *“value that a participant derives from gaining acceptance and approval of the other participants, and improving their social status within the community because of their contribution”* (p.244). Studies by Ifinedo (36), Raza et al. (40), and Cheung et al. (48) concluded that social enhancement is positively related to social media use. Users will use social media if they feel that it makes them feel important, as well as allowing them to enhance their social life. In light of this, the following hypothesis is proposed:

H4. Social enhancement has a positive relation with the use of social media.

According to Dholakia et al. (39), maintaining interpersonal connectivity is defined as *“social benefits derived from establishing and maintaining contact with other people such as through social support, friendship, and intimacy”* (p.244). Previous studies have found a positive relationship between maintaining interpersonal connectivity and social media use. These include those by Raza et al. (40) and Sheldon et al. (49) on the motives for using Instagram, Oliveira and Huertas (47) and Ellison et al. (50) on Facebook relationship maintenance behavior, and Cheung et al. (48) on the use of Facebook. Users will use social media if they believe that it allows them to connect with others and stay in touch. Therefore, the following hypothesis is proposed:

H5. Maintaining interpersonal connectivity has a positive relationship with the use of social media.

### 2.4 Usage behavior towards social relationships and psychological well-being

There have been several studies conducted on the impact of using social media from the perspective of different domains. In the area of health and well-being, examples include the studies of Popat and Tarrant (51) on adolescents’ perspectives on social media and mental health and well-being, Cotten et al. (13) on social media use and well-being among older adults, and Parry et al. (52) on social media and well-being. However, this study focuses on the impact of social media on two important factors: social relationships and psychological well-being.

According to August and Rook (53), social relationships can be defined as “connections that exist between people who have recurring interactions that are perceived by the participants to have personal meaning. This definition includes relationships between family members, friends, neighbors, coworkers, and other associates.” Social relationships are important because they provide a means for intimacy (54), giving and receiving social support (55), social integration (56), and a sense of belonging (57). While social relationships are important for everyone’s well-being (58), Baym et al. (59) and Blais et al. (60) go further to conclude that there is a positive relation between Internet use and social relationships. After using social media, users tend to be more satisfied with their personal as well as their intimate relationships as well as being able to receive support from their family and friends. In light of this, the following hypothesis is proposed:

H6. The use of social media has a positive relation with social relationships.

Psychological well-being was described by Ryff (61) as a multidimensional model that consists of six psychological dimensions: autonomy, environmental mastery, personal growth, positive relations with others, purpose in life, and self-acceptance. Studies by 2, 13–15, 27, 28, 34, 37, 39, 42, 43, 46–48, 50, 55–57, 59, 60, Bano et al. (62)–Huang (63), and Heo et al. (64) concluded that a positive relationship exists between the use of social media and psychological well-being. In addition, Valkenburg and Peter (65) found a mediating effect between the use of social media and psychological well-being. That is, after using social media, users often feel that they enjoy life more and find it to be more meaningful. They also tend feel better and are more satisfied with themselves. Moreover, they will not feel depressed or anxious. Therefore, the following hypothesis is proposed:

H7. The use of social media has a positive relation with psychological well-being.

**Figure 4:**
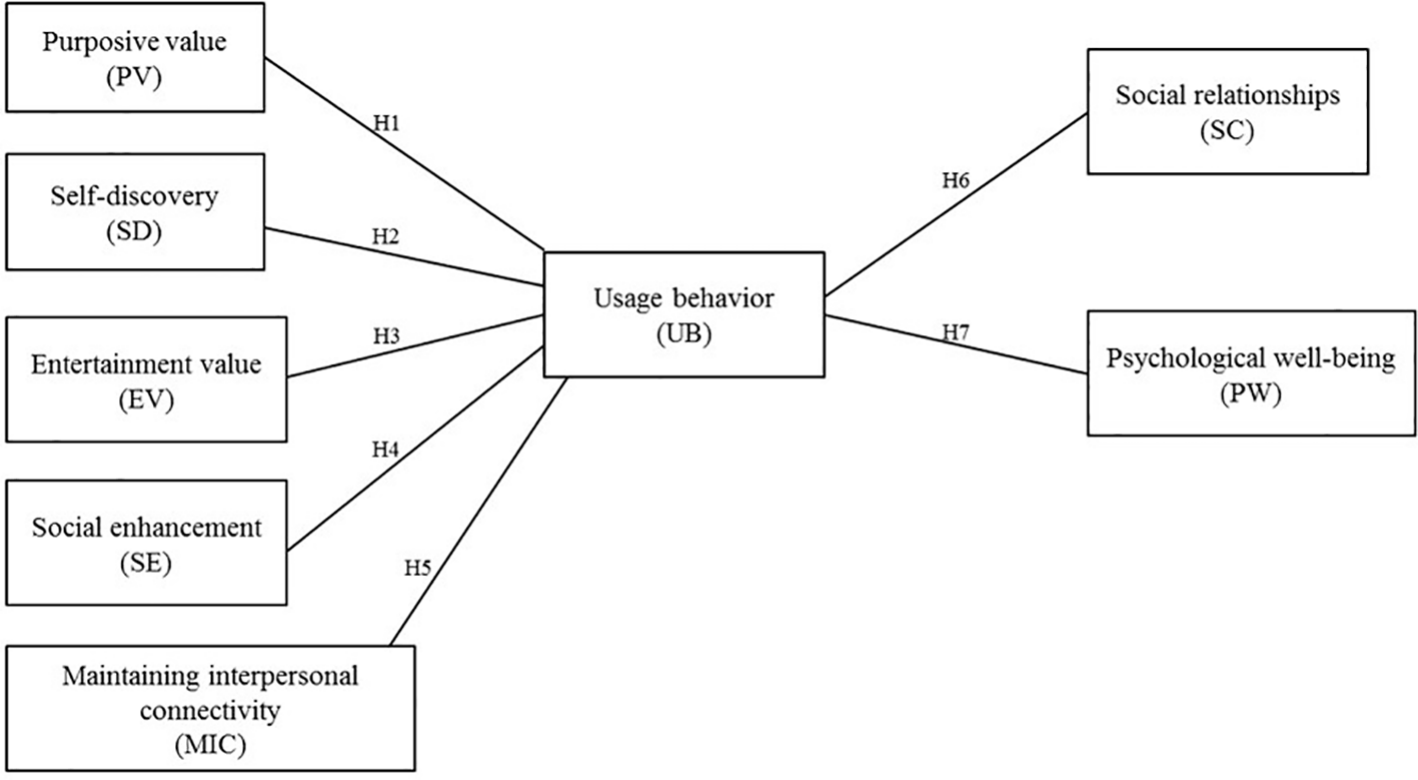
Proposed research model.

The proposed research model and hypotheses are shown in **Figure 4**.

## 3 Research methodology

### 3.1 Instrument development

This study uses a model consisting of the five constructs from the uses and gratification theory (UGT)and their effects on usage behavior and the impacts they have on social connectedness and psychological well-being. Several items were used to measure each construct, and the 7-point Likert scale was used is this study. For the determinants of UGT, the items were modified from Ifinedo (36) and, for usage behavior, from Venkatesh and Davis (66), while the concepts of social relationships and psychological well-being were adapted from the World Health Organization (67).

The questionnaire was initially prepared in English and then translated to Thai for better understanding since Thai is the native language of the participants. The questionnaire was then translated back into English to ensure its accuracy with the original version and only then approved by the Office of the Committee for Research Ethics, Faculty of Social Sciences and Humanities, Mahidol University with the following approval number: 2020/159.0408. The Office of the Committee for Research Ethics is in full compliance with the International Guidelines of Human Research Protection such as the those listed in the Declaration of Helsinki, The Belmont Report, and the CIOMS Guidelines.

Following the ethics approval, a pilot test was performed with thirty participants to ensure that it was clear and easy to understand. The suggestions made by the participants were then used as inputs to modify the questionnaire, which resulted in it being clearer and easier to understand. The questionnaire was made up of two sections: the demographics of the participants and the participants’ usage of social media.

### 3.2 Data collection

Data were collected from participants from two age groups, those between 45–64 and those 65 and older, in order to meet the research objectives of this study. Participants were approached randomly and asked a screening question as to whether they use social media. The participants were approached at three main locations—department stores, hospitals, and public parks in Bangkok—because it was assumed by the researcher and his data collection team that these three locations were the most frequented by people of those two age groups. The participants were given a consent form to sign stating that they consented to participate in the research project.

In total, 1,200 people participated in answering the questionnaire, with 1,176 questionnaires being useful and 24 having to be discarded because of missing data. The participants’ demographics are shown in **Table 2**.

**Table 2:** Demographics of the participants.

Option	Frequency	Percentage
Gender		
Male	506	43.0
Female	670	57.0
Age		
45-49	400	34.0
50-54	285	24.2
55-59	153	13.0
60-64	126	10.7
65-69	161	13.7
Above 70	51	4.3
Education level		
Less than High School	116	9.9
High School/GED	131	11.1
Associate degree	59	5.0
Undergraduate degree	616	52.4
Graduate degree	228	19.4
Doctoral degree	26	2.2
Social media usage		
Under 1 year	33	2.8
1-2 years	85	7.2
2-3 years	118	10.0
3-4 years	207	17.6
Over 4 years	733	62.3
Occupational status		
Paid employment (full-time)	441	37.5
Paid employment (part-time)	30	2.6
Own Business	216	18.4
Retired	213	18.1
Homemaker	99	8.4
Other	177	15.1
Social media services use		
Facebook	283	24.1
Instagram	78	6.6
Line	633	53.8
WhatsApp	2	0.2
YouTube	133	11.3
Twitter	46	3.9
Other	1	0.1
Tool to access social media		
Desktop/PC	34	2.9
Notebook/Laptop	34	2.9
Tablet	43	3.7
Smartphone	1,065	90.6

## 4 Data analysis and results

The two-step approach initially proposed by Anderson and Gerbing (68) was used to analyze the data. The first step is the measurement validity analysis while the second step is the structural model analysis. This was undertaken so that both the proposed hypotheses and the model could be tested.

### 4.1 Measurement validity analysis

Construct reliability as well as validity were conducted in order to test the reliability of the scales. Cronbach’s alpha was used as an indicator. Cronbach’s alpha values were in the range of 0.746-0.930, which were above the acceptable recommended value of 0.70, indicating that the scales were reliable (69, 70). Exploratory factor analysis (EFA) as well as principal component analysis (PCA) and Kaiser’s varimax rotation were also calculated. The KaiserMeyer-Olkin (KMO) returned a value of 0.899, which is above the 0.80 recommended value (71). The results of the EFA are shown in the Appendix.

In addition, the study also conducted a confirmatory factor analysis (CFA). All of the factor loadings except for two items were above the 0.50 threshold value suggested by Hair et al. (70). The two items, purposive value (PV1) and psychological well-being (PW6) were removed from the study because they exhibited low factor loadings. The CFA result is shown in **Table 3**. Furthermore, the composite reliabilities (CR) exceeded the recommended value of 0.50 while almost all the average variance extracted (AVE) exceeded the recommended value of 0.50 except for PV and PW, which were slightly below at 0.495 and 0.450 respectively. However, Fornell and Larcker (72) suggested that if the AVE for that construct is below 0.50 but the CR is above 0.60 then the construct validity is acceptable. Also, as suggested by Fornell and Larcker (72), the square root of the AVEs exceeds the correlations implying good discriminant validity as shown in **Table 4**.

**Table 3:** Item loading on related factors.

Factor	Item	Factor Loading	AVE	CR	Cronbach’s alpha
PV	PV2	0.649	0.491	0.746	0.746
	PV3	0.721			
	PV4	0.738			
SD	SD1	0.857	0.819	0.931	0.930
	SD2	0.907			
	SD3	0.949			
EV	EV1	0.948	0.703	0.903	0.899
	EV2	0.951			
	EV3	0.693			
	EV4	0.727			
SE	SE1	0.525	0.613	0.819	0.785
	SE2	0.929			
	SE3	0.836			
MIC	MIC1	0.742	0.739	0.894	0.884
	MIC2	0.902			
	MIC3	0.923			
UB	UB1	0.713	0.674	0.860	0.860
	UB2	0.867			
	UB3	0.871			
SC	SC1	0.819	0.677	0.863	0.860
	SC2	0.794			
	SC3	0.854			
PW	PW1	0.743	0.450	0.800	0.794
	PW2	0.810			
	PW3	0.671			
	PW4	0.524			
	PW5	0.565			

PV, Purposive value; SD, Self-discovery; EV, Entertainment value; SE, Social enhancement; IC, Maintaining interpersonal connectivity; UB, Usage behaviour; SC, Social relationships; PW, Psychological well-being.

**Table 4:** Correlation coefficient matrix and square root of AVEs.

	PV	SD	EV	SE	MIC	UB	SC	PW
PV	0.704							
SD	0.583	0.964						
EV	0.438	0.362	0.948					
SE	0.401	0.249	0.251	0.886				
MIC	0.442	0.326	0.450	0.316	0.940			
UB	0.530	0.264	0.463	0.288	0.462	0.927		
SC	0.481	0.429	0.488	0.354	0.560	0.391	0.927	
PW	0.541	0.575	0.558	0.510	0.375	0.392	0.684	0.891

### 4.2 Model testing results

**Figure 5:**
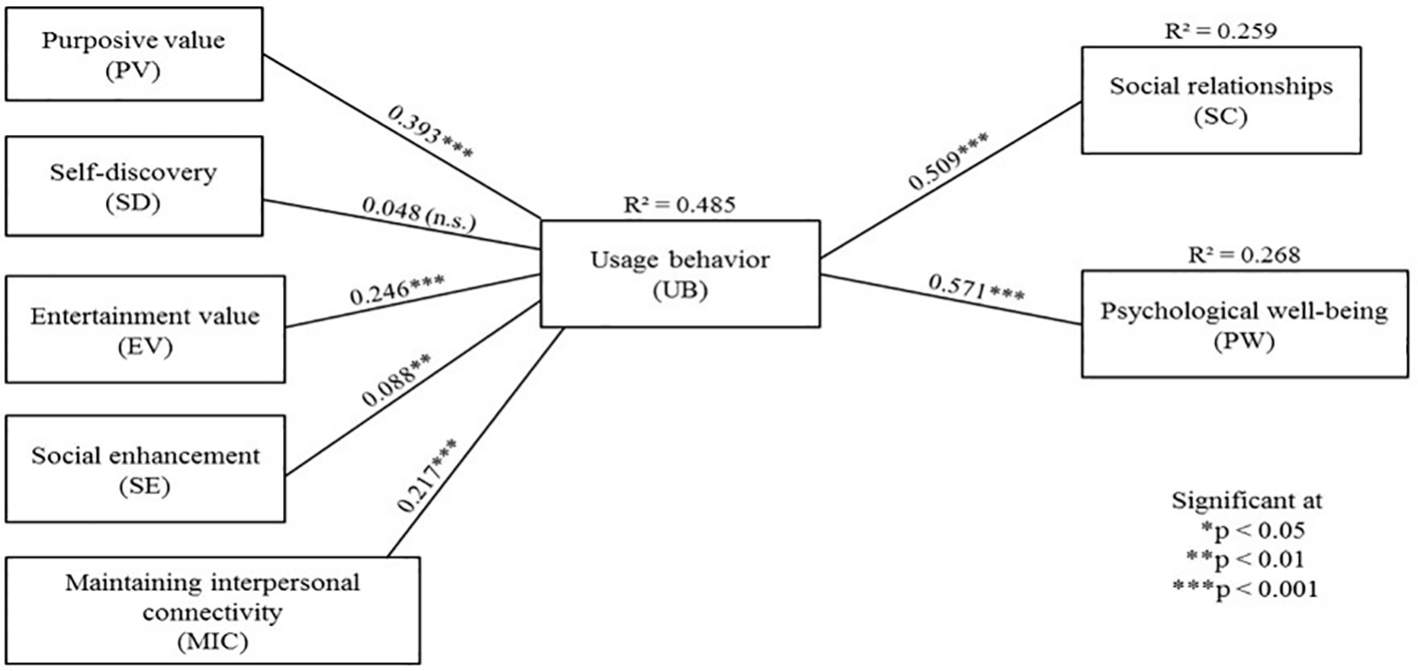
Path diagram and hypotheses testing results.

In order to test the path and the hypotheses proposed in the model, the study analyzed moment structures (AMOS) 24. Gefen et al. (73) have suggested that in order to test structural models and path analysis, AMOS is a good fit. The study used structural equation modeling (SEM) as the main statistical tool in this study because it can perform simultaneous analysis of the relationships between multiple independent and dependent variables, unlike analyzes of variance (ANOVA) or linear regression (68). The fit indices presented in **Table 5** shows that they meet the threshold of the recommended values as recommended in the literature, suggesting a good fit between the data and the model, while the path coefficients are shown in **Figure 5**. The model accounts for 49% of the variance in usage behavior, 26% of the variance in social relationships, and 27% of the variance in psychological well-being. **Table 6** summarizes the hypotheses tests while **Table 7** presents the results of the multi-group analysis. The results of the goodness-of-fit measures for the multi-group analysis is displayed in **Table 8**.

**Table 5:** Goodness-of-fit measures of the research model.

Fit index	Model value	Recommended value
Comparative fit index (CFI)	0.871	>0.80
Goodness fit index (GFI)	0.824	>0.80
Incremental fit index (IFI)	0.908	>0.80
Normal fit index (NFI)	0.858	>0.80
Non-normed fir index (NNFI)	0.865	>0.80
Root mean square error of approximation (RMSEA)	0.074	<0.80

**Table 6:** Hypotheses tests’ summary.

Hypothesis	Relationship	Path coefficients	Support
H1	PV-UB	0.393***	Yes
H2	SD-UB	n.s.	No
H3	EV-UB	0.246***	Yes
H4	SE-UB	0.088**	Yes
H5	IC-UB	0.217***	Yes
H6	UB-SC	0.509***	Yes
H7	UB-PW	0.571***	Yes

*: p <.05 **: p <.01 ***: p <.001.

**Table 7:** Result of multiple-group analysis.

Group	Regression	Coefficient (Below 65)	Coefficient (Above 65)
Age			
	PV-UB	0.372 (t=6.050)**	0.724 (t=5.407)**
	SD-UB	-0.60 (t=-1.343)	-0.21 (t=-0.269)
	EV-UB	0.297 (t=7.325)**	0.267 (t=2.973)**
	SE-UB	0.128 (t=2.013)*	0.389 (t=1.965)*
	MIC-UB	0.300 (t=6.209)**	0.210 (t=2.294)*
	UB-SC	0.412 (t=12.976)**	0.358 (t=6.761)**
	UB-PW	0.313 (t=11.237)**	0.422 (t=8.936)**

*: p < 0.5 **: p < 0.01.

**Table 8:** Multi-group analysis and goodness-of-fit measures.

Fit index	Model value	Recommended value
Comparative fit index (CFI)	0.860	>0.80
Goodness fit index (GFI)	0.804	>0.80
Incremental fit index (IFI)	0.861	>0.80
Normal fit index (NFI)	0.837	>0.80
Non-normed fit index (NNFI)	0.838	>0.80
Root mean square error of approximation (RMSEA)	0.064	<0.80

## 5 Discussions

This research is one of the first to propose UGT to understand the adoption of social media among the elderly and its impact on social connectedness and psychological well-being. Six out of the seven hypotheses proposed were supported in this study. Purposive value was found to be positively related to usage behavior (β = 0.393, p < 0.001), which is similar to Whiting and Williams’ (74) and Park et al.,’s (75) findings. This suggests that the adults and older adults are aware of the purpose of using social media. They use them to get information such as news, horoscopes, and gold prices as well as share this information with friends or family in their circle. This reflects a shift in how information or news is consumed from traditional media such as newspapers and radio to social media.

Contrary to the studies of Ifinedo (36) and Jin et al. (44), self-discovery did not posit a positive relationship towards usage behavior. However, this is similar with Ifinedo (36), Raza et al. (40), Oliveira et al. (46), Oliveira and Huertas (47), and Cheung et al.,’s (48) findings. This finding on self-discovery provides an accurate reflection of older adults since their very mature stage of life means that they do not necessarily need social media to self-reflect or discover themselves. They use social media to communicate with their friends and family, as well as a means to represent themselves rather than discover themselves.

A positive relationship between entertainment value and usage behavior was found (β = 0.246, p < 0.001), which is similar to Ifinedo (36) Raza et al. (40), Oliveira et al. (46), Oliveira and Huertas (47), and Cheung et al.,’s (48) findings. This is not surprising as users of all age groups usually have fun using social media and develop pleasure from using it. During their free time, the adults and older adults use various aspects of social media to keep themselves entertained by chatting, listening to music, and watching video clips. This provides them with a way of relaxation as well as relieving their stress. Entertainment value is a driving factor (76) as well as an important value factor (45) in the use of social media.

Similar to what Ifinedo (36) Raza et al. (40), and Cheung et al. (48) have found, social enhancement was found to have a positive relationship with usage behavior (β = 0.088, p < 0.01). This finding is indeed surprising and not surprising at the same time—surprising because at this stage in their lives, both mature adults and older adults might not feel that it is important to impress others. It would not be surprising if the target group were students, who would be more likely to promote themselves so that they could become more visible to others (40). It would be surprising however, in the case of older adults because they may feel lonely and want to feel important as well as feeling the need to enhance their social life, hence the desire to use to social media.

A positive relationship exists between maintaining interpersonal connectivity and usage behavior (β = 0.217, p < 0.001), which is similar to what Raza et al. (40) Sheldon et al. (49), Oliveira and Huertas (47), Ellison et al. (50), and Cheung et al. (48) found. This finding is not surprising because adults and older adults find it important to be able to connect and keep in touch with family and friends, especially older adults since some of the them might find it difficult to commute or meet friends in person because of health issues. Thus, the use of social media enables them to maintain their relationships at least to some extent. Indeed, Sheldon et al. (49) conclude that the most important gratification factor for the use of social media is maintaining interpersonal connectivity.

The study also found a positive relationship between social media use and social relationships (β = 0.509, p < 0.001). This is likely because social relationships are especially important among older adults as they have smaller social networks compared to younger adults (77). Therefore, in this case they are able to create and maintain social connections through their existing social networks through social media use, which is especially important for adults with health-related or mobility limitations (64). By using social media, they are satisfied with their relationships as well as the support they receive from their social networks.

Consistent with Bano et al. (62) Heo et al. (64) Huang (63), and Valkenburg and Peter (65), social media use was found to be positively related to psychological well-being (β = 571, p < 0.001). From the perspective of Huang (63), psychological well-being is affected by four factors: depression, life satisfaction, loneliness, and self-esteem. Taking that and the findings of the study into account, it can be inferred that by using social media, younger adults and the elderly are more satisfied with their lives and thus find them to be more meaningful. And by using social media, they might exhibit lower levels of depression and loneliness.

Furthermore, the study conducted a multi-group analysis among both younger and older adults. The objective was to test for the differences in the antecedents that led to their usage of social media and their effects on social relationships and psychological well-being. This is important because it provides us an understanding of their behavior, as they will in a few years move into the aging category. The results are similar for both age groups, in terms of the number and type of hypotheses supported. For both groups, the same six hypotheses were supported, with self-discovery being the only one that was not supported. However, the order of the strengths of the relationship were different. For younger adults, the factors that were important in using social media were, in order: purposive value, maintaining interpersonal connectivity, entertainment value, and social enhancement. While for older adults the ranking was: purposive value, social enhancement, entertainment value, and maintaining interpersonal connectivity.

Interestingly, both age groups used social media to fulfill their purposive values whether it was finding information on deals, products, or services. This similar usage also indicates that neither group considers social media merely as an entertainment tool rather a productivity tool. For younger adults, social enhancement was the least important factor (although positive) while for older adults it was maintaining interpersonal connectivity. It is indeed surprising because it would seem that younger adults would be more interested in using social media to enhance their social status or create professional connections, assuming that more of them are still working. As for older adults, maintaining interpersonal connectivity was the least important factor (although positive) indicating that although they use social media to stay in touch with family and friends, it might not be the most important factor. In terms of the impact of social media use, social relationships rather than psychological well-being carry more weight for younger adults. This is expected, as users of this age group tend to socialize and create new relationships. For adults, psychological well-being means more to them compared to social relationships. This is also expected as adults might be more reliant on social media since they are not working/retired, and the use of social media helps them relieve their stress as well as make their life seem more significant.

There are a few limitations in this study. This study was conducted in Thailand and the data was mainly collected only in Bangkok. As Bangkok is the capital and most technologically advanced city in Thailand, the number of people who have access to smartphones, high Internet speeds, and social media will be higher than in other provinces. Future studies should also take into consideration participants from major cities as well as rural provinces to see if there is any difference in the usage behavior and its impact. In addition, the study here employs only the uses and gratification theory; perhaps other factors that were not included in this study could be undertaken to provide a more complete view of social media use and its influence. Lastly, the study used a cross sectional method in collecting data. The use of longitudinal data could possibly provide better results.

## 6 Conclusion

This study proposed an extensive model consisting of constructs from UGT, usage behavior, social relationships, and psychological well-being in order to identify the degree of influence each construct has on social media adoption and the effects it has on the social relationships and psychological well-being among adults below and above age 65. An extensive review of the literature as well as a field survey were undertaken. Of the seven hypotheses proposed, six were accepted; self-discovery as a motive for usage was the only one that was not accepted. Purposive value had the highest impact on social media use, while the use of social media had more impact on psychological well-being when compared to social relationships. The study also conducted a multi-group analysis among adults both below and above age 65. For both groups, self-discovery was the only factor that did not have a positive relationship towards social media use, while for both genders purposive value was the most important factor towards social media use. For younger adults the impact of social media was higher for social relationships compared to psychological well-being whereas for older adults the impact of social media use was higher for psychological well-being when compared to social relationships.

With regard to academic contributions this study adds new findings to the body of knowledge. Although the uses and gratification theory has been applied in previous studies, it has not been tested for social media adoption and its impact on social relationships and psychological well-being, neither has it been tested in an emerging economy such as that of Thailand. Compared to previous studies, this study presents consistencies as well as contradictions in its findings. This is indeed interesting as it proves that models developed in Western countries might be replicated but will not yield similar results. This is why it is important to test them in different contexts and cultures so that they can serve as a guide and can be replicated in countries that have similar contexts, cultures, or even technological advancement.

With regard to practical contributions, the study provides solid empirical evidence on social media usage and the impact that it has on adults, especially older adults. This is important from both the private as well as the public sector perspective. From the private sector perspective, this study provides an insight to social media applications developers so that they can better understand the needs of the elderly. Perhaps in the future they could even develop applications that solely serve as a platform for older adults to connect because some of the current social media applications are not entirely developed with adults or, especially, older adults in mind as the primary demographic.

From the public sector perspective, related agencies or ministries such as the Ministry of Social Development and Human Security can develop training classes for older adults. The classes can focus on improving their technical skills as well as introduce them to different social media platforms and their benefits. Conducting classes will also have an indirect impact as older adults will also have an opportunity to make new friends during the class with whom they can continue to stay in touch through social media. As evident from this study, social media use has a positive impact on the social relationships and psychological well-being of both younger and older adults. Moreover, public and private hospitals could also provide support for all social media users, perhaps in the form of a hotline or physical centers that provide counseling or advice to those that overuse social media. Studies have shown that the use of social media also has negative impacts such as anxiety, depression, and lack of sleep. For adults that are not able to travel to hospitals or for whom using the hotline is not convenient, home care providers for seniors could perhaps assist them. In addition to health-related issues adults should also be made aware of scams and frauds that they might encounter while using social media.

Based on the above it can be clearly seen that this study addresses a few gaps in literature. It is evident that this topic has received a lot of interest among researchers. However, an extensive literature review has found no studies measuring the impact on social relationships and psychological well-being, especially in the context of an emerging economy like Thailand. Previous studies focused on understanding the use of social media and one age group, such as adolescents or the elderly. However, this study performed a multi-group analysis of both adults as well as older adults which provides an insight into the impact that social media has on their social relationships and psychological well-being.

## Data availability statement

The raw data supporting the conclusions of this article will be made available by the authors, without undue reservation.

## Ethics statement

The studies involving humans were approved by Office of the Committee for Research Ethics, Faculty of Social Sciences and Humanities, Mahidol University with the following approval number: 2020/159.0408. The studies were conducted in accordance with the local legislation and institutional requirements. The participants were given a consent form to sign stating that they consented to participate in the research project.

## Author contributions

VB: Conceptualization, Formal analysis, Funding acquisition, Investigation, Methodology, Visualization, Writing – original draft, Writing – review & editing.

## Footnotes

1 Adults are those that are 18 years or older while older adults are 65 years and older as designated by the American Medical Association (AMA).

## Appendix

### Exploratory component analysis (EFA)

**Table:**

	Component							
	PV	SD	EV	SE	MIC	UB	SC	PW
PV1	.739							
PV2	.744							
PV3	.629							
SD1		.855						
SD2		.875						
SD3		.888						
EV1			.882					
EV2			.875					
EV3			.724					
EV4			.758					
SE1				.419				
SE2				.898				
SE3				.871				
MIC1					.738			
MIC2					.872			
MIC3					.866			
UB1						.820		
UB2						.854		
UB3						.824		
SC1							.795	
SC2							.841	
SC3							.727	
PW1								.458
PW2								.623
PW3								.503
PW4								.591
PW5								.801
